# In Vitro Evaluation of Anti-Hemolytic and Cytotoxic Effects of Traditional Mexican Medicinal Plant Extracts on Human Erythrocytes and Cell Cultures

**DOI:** 10.3390/life14091176

**Published:** 2024-09-18

**Authors:** Joel H. Elizondo-Luevano, Ramiro Quintanilla-Licea, Sandra L. Castillo-Hernández, Eduardo Sánchez-García, Minerva Bautista-Villarreal, Georgia M. González-Meza, Marcela A. Gloria-Garza, Osvelia E. Rodríguez-Luis, Maciej Ireneusz Kluz, Miroslava Kačániová

**Affiliations:** 1 Faculty of Biological Sciences, Universidad Autónoma de Nuevo León, Cd., San Nicolás de los Garza 66455, Nuevo León, Mexico; ramiro.quintanillalc@uanl.edu.mx (R.Q.-L.); sandra.castilloh@uanl.mx (S.L.C.-H.); eduardo.sanchezgrc@uanl.edu.mx (E.S.-G.); minerva.bautistavl@uanl.edu.mx (M.B.-V.); 2 Faculty of Pharmacy, University of Salamanca, 37008 Salamanca, Spain; 3 School of Engineering and Sciences, Tecnológico de Monterrey, Monterrey 64849, Nuevo León, Mexico; georgia.gonzalez@tec.mx; 4 Faculty of Odontology, Universidad Autónoma de Nuevo León, Dr. Eduardo Aguirre Pequeño, Monterrey 64460, Nuevo León, Mexico; marcela.gloriagz@uanl.edu.mx (M.A.G.-G.); osvelia.rodriguezls@uanl.edu.mx (O.E.R.-L.); 5 School of Medical & Health Sciences, University of Economics and Human Sciences in Warsaw, Okopowa 59, 01 043 Warszawa, Poland; m.kluz@vizja.pl; 6 Institute of Horticulture, Faculty of Horticulture and Landscape Engineering, Slovak University of Agriculture, Tr. A. Hlinku 2, 94976 Nitra, Slovakia

**Keywords:** AAPH, antioxidant activity, DPPH, erythrocytes, ethnopharmacology, hemolysis, herbal medicine, PBMC, Vero cells

## Abstract

Plant extracts of fifteen plants of ethnomedicinal use in Mexico were analyzed to provide scientific knowledge of their medicinal properties through the evaluation of different biological activities such as anti-hemolytic, antioxidant, and cytotoxic effects in normal cells. Therefore, methanolic extracts were obtained from each of the plants by the Soxhlet extraction. The hemolytic activity in human erythrocytes was evaluated, as was their potential to protect the erythrocyte membrane against the 2,2′-azobis (2-methylpropionamidine) dihydrochloride (AAPH) and 1,1–diphenyl–2–picryl hydrazyl (DPPH) radicals. Finally, the toxicity of the extracts in normal cell cultures of African green monkey kidney cells (Vero) and peripheral blood mononuclear cells (PBMC) was determined by the 3-(4,5-dimethylthiazol-2-yl)-2,5-diphenyltetrazolium bromide (MTT) reduction method. Most of the extracts showed low hemolytic activity and high anti-hemolytic activity as well as high selectivity indices (SI) and antioxidant effects. Extracts of *H. inuloides*, *J. dioica*, and *J. spicigera* induced cell proliferation of the Vero cells. *K. daigremontiana*, *A. adstringens*, *S. mexicanum*, *J. spicigera*, *L. tridentata*, and *M. tenuiflora* extracts showed PBMC cell proliferation. In the present study, it was observed that the evaluated extracts did not present hemolytic activity, and some presented low toxicity when Vero and PBMC cell cultures were exposed. In conclusion, traditionally used plants possess beneficial health properties, and it is hoped that this study will serve as a basis for understanding the biological effects of traditionally used plants and may complement future studies.

## 1. Introduction

Plants represent an important source of metabolites with diverse biological properties that can be used as active ingredients for the treatment of diseases [1,2]. The use of medicinal plants dates to ancient times, and the World Health Organization (WHO) recognizes their important value [3]. In recent years, it has been shown that plant secondary metabolites represent a source of potent biological agents [4,5]. Due to its biogeographic position, Mexico has a great plant biodiversity, and millions of people use traditional medicines [6]. There are more than 4500 plants in the country that have been traditionally used to treat various ailments, such as infectious diseases and cancer [7].

Some commonly used and specialized drugs have been identified and extracted from plants [8]. Some examples are phytohormones extracted from the plants striga and orobanche [9], the antineoplastics Paclitaxel from the bark of *Taxus brevifolia*, the alkaloid vincristine extracted from the plant vinca, the cardiotonics digoxin, digitoxin, and digoxigenin present in the phanerogam plant *Digitalis lanata*, *Digitalis purpurea*, and *Digitalis orientalis* used as anti-arrhythmic agents for heart failure [10]. However, the uncontrolled use of some plants, posologies, and infusions could cause toxic effects for those who consume them [11]. Because of that, it is important to evaluate their toxic potential or the safe doses [12].

The selection of plants for this study was guided by their traditional use in Mexican ethnomedicine and their reputed beneficial properties [6]. Traditional Mexican medicinal plants have been utilized for centuries in various cultural practices due to their perceived health benefits, including anti-hemolytic and cytotoxic effects [13]. This long history of use provides a strong rationale for investigating their biological activities using modern scientific methods. The plants chosen for this study were selected based on their traditional significance and documented ethnobotanical uses, ensuring a diverse representation of plant species known for their medicinal properties [13]. By evaluating these traditionally used plants, this study aims to bridge traditional knowledge with contemporary scientific research, thereby validating and potentially expanding their therapeutic application.

The hemolysis test represents a basic biological toxicity test commonly used to evaluate the activity of extracts or natural products [14] because this test is rapid, reproducible, and inexpensive compared to some tests such as cell culture. Although human erythrocytes are an option for the evaluation of preliminary in vitro toxicity testing of natural products intended for human use [15], it is not uncommon to use erythrocytes from other animal species for such evaluation [16,17].

The use of cell cultures in the validation of traditional medicinal plant extracts is essential for accurately and systematically evaluating their biological properties [18]. Cell cultures allow for the examination of how extracts impact cell viability and functionality in a controlled environment that mimics specific biological conditions [19]. This methodology is crucial because it provides a platform to investigate the effects of extracts at the cellular level, helping to confirm the beneficial properties reported in traditional studies [20]. Phytocomponents, such as flavonoids and phenolics, play a significant role in neutralizing free radicals and reducing oxidative stress [21]. By evaluating these extracts, this study aims to highlight their potential in protecting cells from oxidative damage and to explore their therapeutic applications. Additionally, cell culture studies facilitate the identification of potential mechanisms of action and side effects, ensuring a more comprehensive assessment of their therapeutic potential [22].

There are several examples in the literature in which erythrocytes and cell cultures in in vitro assays have served as a model [19,20] for the study of the biological activity of natural products [23] and medicinal plant extracts [24]. In this context, the main objective of this study was to evaluate the in vitro hemolytic/anti-hemolytic potential in human erythrocytes of methanolic extracts of some plants of traditional use in Mexico, as well as their antioxidant effect to determine their toxicity in a cellular model on normal Vero (monkey kidney epithelial cells) [25] and PBMC (human peripheral blood mononuclear cells) [26] cells and to provide an overview of the concentrations of their safe use.

## 2. Materials and Methods

### 2.1. Cells

The monkey kidney epithelial cells (Vero; ATCC^®^ CCL-81™) were obtained from the American Type Culture Collection (ATCC^®^, Manassas, VA, USA). The human peripheral blood mononuclear cells (PBMC), and human erythrocytes were provided by the Faculty of Medicine of the Universidad Autónoma de Nuevo León (UANL).

The Vero cell line was cultured in Dulbecco’s modified Eagle’s medium (DMEM, Gibco, Grand Island, NY, USA). The tests performed with Vero cells were carried out in 96-well, flat-bottom plastic microplates (Corning^®^ Labware and Equipment, New York, NY, USA) due to the adherent nature of these cells [27]. The PBMC cells were maintained in Roswell Park Memorial Institute medium (RPMI-1640, Sigma-Aldrich^®^, Merck KGaA, Darmstadt, Germany). The tests performed with PBMC cells were carried out in 96-well, curved-bottom plastic microplates (Corning^®^) because these cells are non-adherent. The cells were maintained within a humidified incubator with 5% CO_2_ at 37 °C, cultured and supplemented with 10% fetal bovine serum (FBS, Biosharp, Tallinn, Estonia) and 1% antibiotic/antimycotic (Gibco). The medium was replaced every 48 h. When the cell density reached 80% or above, trypsin (Beyotime Biotechnology, Shanghai, China) was used for digestion and passage [28].

### 2.2. Plant Material

The plants used in this study were identified and deposited at the herbarium of Facultad de Ciencias Biológicas (FCB) at UANL. Each plant was provided with a voucher number from the FCB-UANL herbarium. The plants were acquired from a certified supplier of medicinal plants in Mexico (Pacalli^®^ Herbolaria Científica, Guadalupe, N.L., Mexico), which guarantees their authenticity and quality. The taxonomy of the plants has been validated on the World Flora Online (WFO) website (www.worldfloraonline.org, accessed on 3 August 2024) and on the International Plant Names Index (IPNI) website (https://www.ipni.org/, accessed on 3 August 2024).

### 2.3. Extraction

For each plant, 100 g of milled dry material was treated with 1.0 L of absolute methanol (MeOH, CTR^®^ Scientific, Monterrey, NL, Mexico) by the Soxhlet method for 72 h [15]. These are the crude methanol extracts. The extracts were filtered using a grade 1 filter paper (Whatman™, Global Life Sciences Solutions USA LLC, Marlborough, MA, USA) and were rota evaporated in a Yamato RE200 rotary evaporator (Yamato Scientific Co., Ltd., Harumi, Chuo-ku, Tokyo, Japan) at 100 rpm/40 °C in a water bath [29]. The yields were calculated with the Formula (1):

(1) $$Yield\;\%=\frac{Final\;weight}{Initial\;weight}\times 100$$

### 2.4. Preliminary Phytochemical Screen

The crude extract underwent a phytochemical screening process. These tests were reported as presence (+) and absence (−) and included the following tests: Dragendorff (alkaloids), anthrone (carbohydrates), sodium hydroxide (coumarins), Shinoda (flavonoids), Bornträger (quinones), sodium bicarbonate (saponins), Baljet (sesquiterpene lactones), Liebermann-Burchard (sterols and triterpenes), and ferric chloride (tannins) [30].

### 2.5. Hemolytic Activity

Hemolytic activity was evaluated using the hemolysis test [31]. Treatments were prepared in phosphate-buffered saline (PBS 1× at pH 7.4) and tested at concentrations of 125.5, 250, 500, and 1000 µg/mL (*w*/*v*). Hemolysis was quantified by measuring absorbance (Abs) at 540 nm for each treatment. The assays were performed in 96-well, round-bottom microplates (Corning^®^ Labware and Equipment, Oneonta, NY, USA). Hemolysis of human red blood cells was calculated using the following Formula (2):

(2) $$Hemolysis\;\%=\frac{{Abs}_{540\mathrm{nm}}\;Treatment}{{Abs}_{540\mathrm{nm}}\;Positive\;control}\times 100$$

### 2.6. Anti-Hemolytic Activity

The 2,2′-azobis(2-methylpropionamidine)dihydrochloride (AAPH) inhibition test, as previously described [32], was employed to assess anti-hemolytic activity. Hemolysis was induced by the AAPH radical (150 mM) as a positive control (Sigma-Aldrich^®^, Merck KGaA, Darmstadt, DE, Germany). Treatments were tested at concentrations ranging from 20 to 200 µg/mL (*w/v*) alongside AAPH. The assays were performed in 96-well, round-bottom microplates (Corning^®^). The anti-hemolytic effect was calculated using the following Formula (3):

(3) $$Anti-hemolytic\;Activity\;\%=100-\left(\frac{{Abs}_{570\mathrm{nm}}\;Treatment}{{Abs}_{570\mathrm{nm}}\;Positive\;control}\times 100\right)$$

### 2.7. Selectivity Index

Selectivity indices (SI) were determined as follows (4) [33]:

(4) $$SI=\frac{{IC}_{50}\;Hemolytic\;Activity}{{IC}_{50}\;Anti-hemolytic\;Activity}$$

### 2.8. Antioxidant Activity

Antioxidant activity was evaluated using the 2,2-diphenyl-1-picrylhydrazyl (DPPH) test [34], with results expressed as IC_50_ values (µg/mL), representing the concentration needed to reduce the initial DPPH concentration by 50%. Vitamin C (Sigma-Aldrich^®^) served as a positive control. The assay was conducted in 96-well microplates (Corning^®^), and DPPH inhibition at 517 nm was calculated using Formula (5):

(5) $$DPPH\;scavenging\;\%=\frac{{Abs}_{517}\;Control-{Abs}_{517}\;Sample}{{Abs}_{517}\;Control}\times 100$$

### 2.9. Cell Viability

The cells were treated with different final concentrations of the extracts (62.5, 125, 250, and 500 µg/mL), solubilized in dimethyl sulfoxide (DMSO; Sigma-Aldrich^®^). The final concentration of DMSO in the assays was less than 0.5% (*v*/*v*), a level that does not affect cell viability [35]. After 72 h of incubation, cell viability was assessed by measuring the Abs at 570 nm using a microplate reader (Thermo Fisher Scientific Inc., Waltham, MA, USA). Cell viability was determined using the 3-(4,5-dimethylthiazol-2-yl)-2,5-diphenyltetrazolium bromide (MTT) colorimetric assay, by adding 15 µL of MTT (500 µg/mL) to each well and incubating for 3 h [36]. The plates were then decanted, and the Formazan crystals were dissolved with 80 µL of DMSO. The control consisted of only the culture medium. Cell viability was calculated using the following Formula (6):

(6) $$Cell\;viability\;\%=\frac{\;{Abs}_{570\mathrm{nm}}\;Treatment}{\;{Abs}_{570\mathrm{nm}}\;Negative\;control}\times 100$$

### 2.10. Statistical Analysis

A one-way variance analysis (ANOVA) followed by Tukey’s test was employed to identify statistically significant differences among the values and was conducted using the SPSS Statistics software package (Version 22.0, IBM^®^, Chicago, IL, USA). The half-maximal inhibitory concentration (IC_50_) values were determined by the Probit test, using the AAT Bioquest IC50 Calculator tool (AAT Bioquest, Inc., Pleasanton, CA, USA).

## 3. Results and Discussion

### 3.1. Taxonomic Identification and Phytochemical Screening

Taxonomic identification of the plants in Table 1 was carried out, and the corresponding methanolic extracts were prepared. The traditional names and the parts used to prepare the extracts of each one of these are also shown. Finally, the extraction yields are reported, which varied between 9 and 38%. The taxonomy of these plants was compared and validated with the WFO and IPNI websites.

Methanol was chosen due to its ability to extract a wide range of bioactive compounds, including secondary metabolites that may not be effectively extracted with aqueous solvents [37]. Additionally, MeOH facilitates higher solubility of many polar and semi-polar compounds present in plants, which can provide a more comprehensive understanding of their biological potential [38].

Table 2 shows the results of the phytochemical tests obtained for each extract. All extracts were positive for instaurations (double bonds) and coumarins. For the alkaloid test, only *K. daigremontiana*, *A. mexicana*, and *R. chalepensis* were positive. Only the extracts of *J. spicigera*, *S. mexicanum*, *S. aspera*, and *T. lucida* were negative for the sterol test. Different parts of a plant, such as leaves, stems, and roots, often contain varying concentrations and types of phytocomponents [37]. Leaves may be rich in flavonoids and antioxidants due to their role in photosynthesis and protection against environmental stress. Stems might accumulate saponins and alkaloids related to structural support and pathogen resistance, while roots are known for storing compounds like alkaloids and terpenes that contribute to soil adaptation and defense.

### 3.2. Hemolytic Activity and Anti-Hemolytic Activity

In this study, we aimed to determine the hemolytic and anti-hemolytic effects of crude extracts in vitro from various traditionally used plants in Mexico and evaluate their antioxidant activity and toxicity in in vitro cell cultures. The objective is to provide and expand knowledge on the ethnopharmacological effects of these traditionally used plants [13].

**Figure 1:**
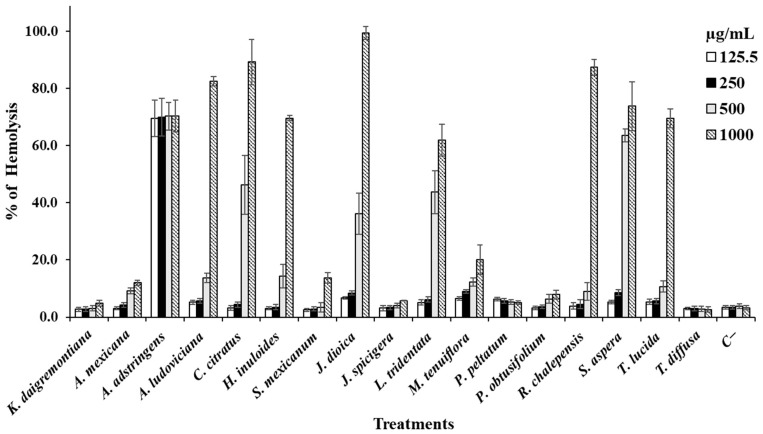
Data are shown as mean ± SD of the hemolysis percentage caused by each extract.

Figure 1 presents the data from the hemolytic activity test of the MeOH extracts on erythrocytes. Several extracts demonstrated no hemolytic effects compared to the positive control, distilled water, which caused complete hemolysis. However, extracts from *A. ludoviciana*, *C. citratus*, *J. dioica*, *R. chalepensis*, and *S. aspera* exhibited hemolytic effects at the highest concentration tested (1000 µg/mL). Conversely, the other extracts showed minimal activity against erythrocytes, with some, like *K. daigremontiana*, displaying effects similar to the positive control across all concentrations tested (*p* < 0.05).

**Figure 2:**
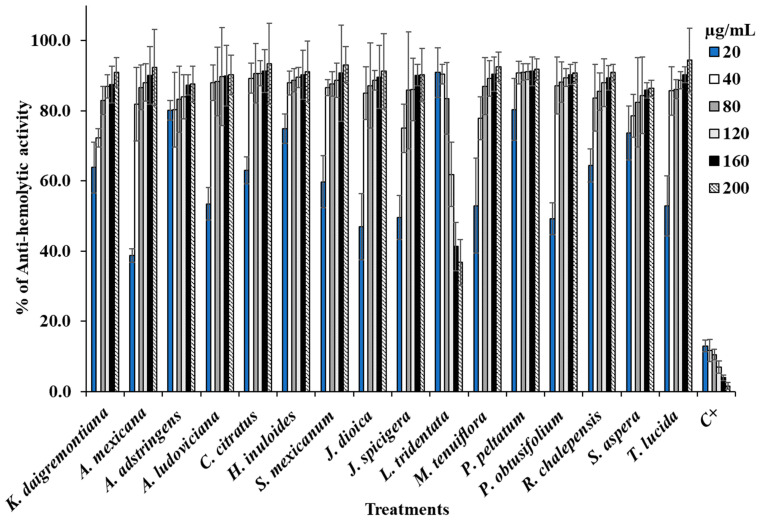
Data are shown as mean ± SD of the anti-hemolytic activity percentage caused by each extract.

In the anti-hemolytic activity tests of methanol extracts on human erythrocytes, Figure 2 shows the hemoprotective effects of several extracts compared to the positive control, the oxidizing agent AAPH, which caused 100% hemolysis. All evaluated extracts demonstrated a dose–response effect, with protective activity increasing alongside extract concentration. However, *L. tridentata* was an exception, as its anti-hemolytic activity decreased with increasing concentrations.

Table 3 presents the IC_50_ values and selectivity indices (SI) for each evaluated extract. The hemolytic effects on human red blood cells revealed IC_50_s ranging from 182.87 µg/mL (*A. adstringens*) to over 1500 µg/mL (*S. mexicanum*, *J. spicigera*, *M. tenuiflora*, *P. peltatum*, and *P. obtusifolium*). For anti-hemolytic activity, the extracts exhibited potent effects. Also, all the extracts showed the highest selectivity indices (SI).

Hemolytic capacity refers to the ability of a substance, such as a natural product extract, to cause hemolysis in erythrocytes, as well as to prevent hemolytic effect against a known hemolytic agent, such as hydrogen peroxide (H₂O₂), Triton X-100, or AAPH, which would indicate its potential as an antioxidant [39,40,41,42]. This type of study is relevant in the investigation of natural antioxidants and other bioactive compounds that could have therapeutic applications in diseases where hemolysis is a critical factor, such as in certain anemias, as well as in the use of possibly toxic products, such as the excessive use of some treatments, or some natural products with toxic potential [14,24,42]. These studies are key to identifying natural compounds that could be used as preventive or adjuvant therapies in the treatment of hemolytic diseases [20,39]. In addition, plants or natural products are used to explore new antioxidants that protect red blood cells from oxidative damage [24,43,44].

Hemolysis studies are part of the preclinical evaluation of new herbal drugs, ensuring their safety before use in humans [30,45,46]. A lower percentage of hemolysis in the presence of the natural product indicates a higher anti-hemolytic capacity [15,46].

### 3.3. Antioxidant Activity Assay

**Figure 3:**
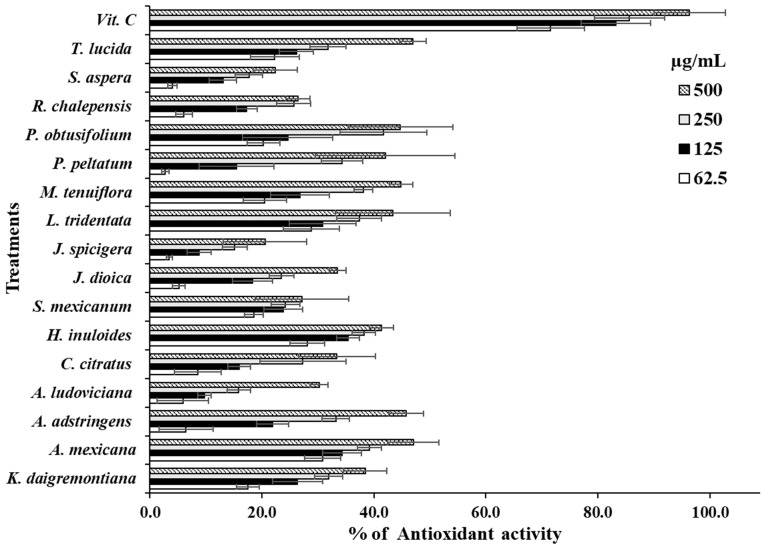
Data are shown as mean ± SD of the antioxidant activity percentage caused by each extract.

Figure 3 shows the results corresponding to the antioxidative activity determined by the DPPH test. The extracts showed a dose–response behavior in which, as the concentration of the extracts increases, their capacity to capture the DPPH radical increases. However, compared with the positive control, vitamin C was more effective than the extracts. Table 4 displays the IC_50_ results obtained from the antioxidant activity assessment. The extracts did not demonstrate greater antioxidant activity compared to the positive control. The extracts with the highest antioxidant activity were *M. tenuiflora*, *P. peltatum*, *P. obtusifolium*, and *T. lucida* showing IC_50_’s = 547.66, 520.52, 528.67, and 578.62 µg/mL, respectively (Table 4).

The antioxidant activity of plants can vary based on the plant part used and its maturity stage, which influences the phenolic content and the efficacy in scavenging DPPH radicals [47]. DPPH radical assays are widely regarded as a reliable method for assessing free radical scavenging ability [48].

Many therapeutic herbs have been used as natural antioxidants, including the *Kalanchoe* species that have strong antioxidant activity. Plant extracts and naturally occurring substances with high antioxidant activity have been shown to be effective in preventing several types of cancers. However, the use of antioxidant medicines as an adjuvant cancer therapy remains contentious due to inconsistent research results [49]. Previous research has revealed the antioxidant capabilities of different species of *Kalanchoe*, but the findings differed from our study [50,51,52]. In a different investigation, the amount of sample required to reduce the DPPH concentration for *A. mexicana* by 50% was shown to be the median effective concentration (EC_50_), that is the point at which a treatment generates a median response following an exposure period [32]. In another study, the scavenging activity of the various aboveground *A. annua* and *A. absinthium* samples was examined by Bordean et al. (2023). Using the DPPH technique, the researchers found that *A. absinthium* leaf extracts had the strongest antiradical activity [53].

The antioxidant capacity of medicinal plants is greatly increased by phenolic compounds, according to several previous researches [54,55,56]. Phenolic compounds such as quercetin, catechins, gallic acid, and resveratrol present in medicinal plants are known for their potent antioxidant activity [21,49,57]. They act by neutralizing free radicals through the donation of electrons or hydrogen atoms and can chelate metal ions that promote the formation of these radicals. These compounds not only protect against oxidative damage, but may also offer benefits in the prevention of diseases related to oxidative stress [57].

### 3.4. Cytotoxic Activity

**Figure 4:**
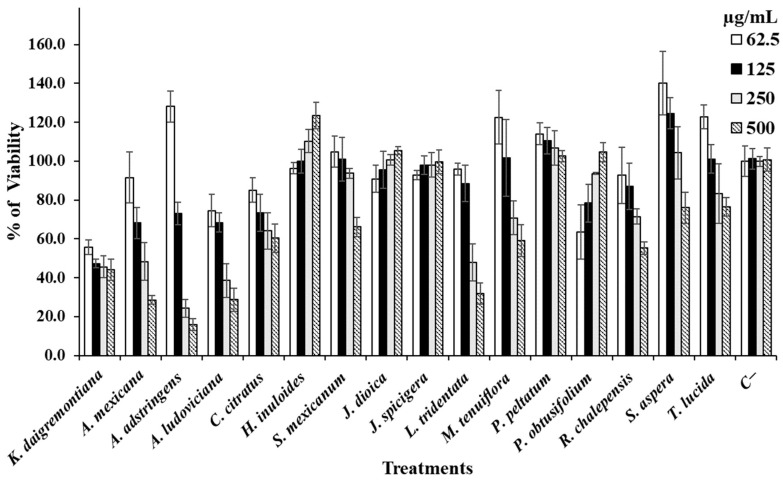
Data are shown as mean ± SD of the viability percentage of Vero cells caused by each extract.

**Figure 5:**
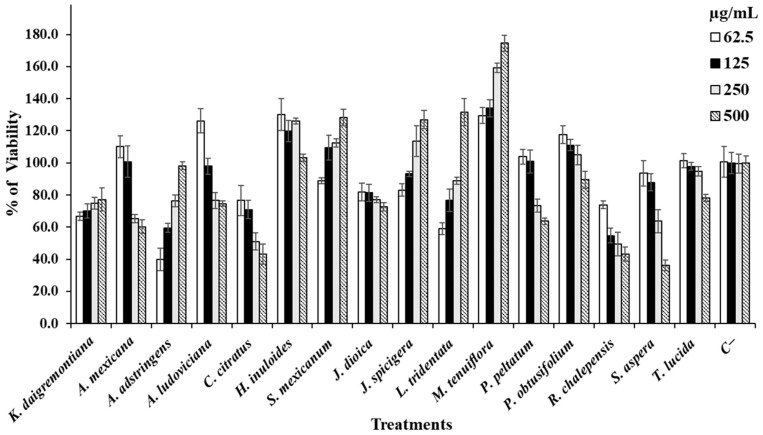
Data are shown as mean ± SD of the viability percentage of PBMC cells caused by each extract.

In addition to the determination of hemolytic and anti-hemolytic activity, the effect of the extracts on healthy cell cultures was determined using Vero and PBMC cells as study models (Figure 4 and Figure 5). The corresponding IC_50_s were also determined (Table 5).

When the extracts were evaluated on Vero cell cultures, it was observed that *H. inuloides*, *J. dioica*, *J. spicigera*, *P. peltatum*, and *P. obtusifolium* induced proliferation of these cells. The other extracts induced a reduction in cell viability between the concentrations of 250 and 500 µg/mL (Figure 4). Figure 5 shows the effect of the extracts against PBMC cultures where it is observed that the extracts of *K. daigremontiana*, *A. adstringens*, *S. mexicanum*, *J. spicigera*, *L. tridentata*, and *M. tenuiflora* induced cell proliferation at all tested concentrations. *P. peltatum* and *P. obtusifolium* caused a slight decrease in cell proliferation at concentrations of 250 and 500 µg/mL, respectively. *T. lucida* showed a slight decrease in viability at 500 µg/mL, and the other extracts caused a decrease in PBMC viability at 500 µg/mL; however, the extracts of *C. citratus*, *S. aspera*, and *R. chalepensis* were the extracts with the worst performance in terms of PBMC viability, as they showed ascending inhibitory activity as we increased the concentrations evaluated.

Table 5 shows the results of the IC_50_ of each of the methanolic extracts on the Vero and PBMC cells. In cases where the extracts promoted high cell proliferation, the corresponding IC_50_s were not calculated (ND).

In our study, the extracts from *K. daigremontiana*, *A. adstringens*, *S. mexicanum*, *J. spicigera*, *L. tridentata*, and *M. tenuiflora* were evaluated against PBMC cultures, and it was found that at all tested concentrations, the extracts caused cell growth. Anacardic acids have been found in the bark before [58]. These compounds were found to be cytotoxic to a number of cell lines at concentrations between 10 and 40 μM (3.4 and 13.6 μg/mL) [59,60]. Since the cytotoxicity rose as the solvent’s polarity decreased, it stands to reason that these lipophilic anacardic acids are also the cytotoxic principle for the Caco-2 cell line. Additionally, Rodriguez–Garcia et al. (2015) discovered that a methanolic bark extract with concentrations ranging from 4.4 to 28.0 μg/mL has antiproliferative effects on human cell lines, including OVCAR-3, UACC-62, HT-29, PC-3, U251, NCI-H460, and 786-O [61]. Anacardic acids, on the other hand, have been demonstrated by Xiu et al. (2014) to stimulate cell migration through the creation of lamellipodia, prevent late apoptosis, and accelerate the proliferation of ovarian cancer cells [62]. Widely used as a medicinal plant, *A. adstringens*, also known as cuachalalate in Mexico, is particularly effective in treating ulcers, cancer, gastritis, and other gastrointestinal ailments as well as accelerating wound healing [63]. Anacardic acids, which are included in plant bark extracts, were found to be cytotoxic to a number of cell lines, but other researchers saw proliferative effects. Furthermore, rats treated with extracts containing masticadienonic acid and its 3α-hydroxy derivative showed anti-inflammatory and anti-ulcer properties [64,65].

In order to ascertain the selectivity of the plant extracts, we assessed their cytotoxic effect on the Vero cells [66] and the PBMC cells [22] because those cells are simple to keep in culture, and they are frequently employed in cytotoxicity investigations [45,67]. The Vero cell line, the first CCL (continuous cell line) approved by the WHO for human vaccine production [68], is non-tumorigenic at low passage numbers and safe for use in vaccines [69]. They help assess pharmacological vaccines [69,70]. The Vero cell line is favored for its ease of handling and its ability to self-replicate indefinitely with a high degree of uniformity. This advantage makes it preferable to primary liver cells, which have a limited lifespan and eventually die after a finite number of generations [71].

PBMCs are representative of circulating immune cells in the body, including T and B lymphocytes, NK cells, and monocytes [72]. Assessing toxicity in these cells helps to anticipate possible adverse effects that a natural extract or compound might have on the immune system [73]. Assessing toxicity in PBMC is an early step in the development of new plant-based treatments, as it allows the identification of potential cytotoxic effects prior to more complex studies in animal models or humans [73,74].

Methanol extract from medicinal plants had a mitogenic effect on the dose- and time-dependent patent in a different study [75]. In this research, the human PBMCs and Vero cells were stimulated to proliferate by some extracts. The above indicates a mitogenic effect of some of the extracts, which is an important property that may indicate the potential of these compounds to modulate the immune system [76,77]. However, it is critical to balance the benefits with the potential risks, ensuring that compounds that promote cell proliferation do so in a safe and controlled manner, without inducing adverse effects such as toxicity or autoimmunity [78,79].

Variability between cell lines can significantly impact the interpretation of results on cytotoxicity and cell proliferation due to inherent differences in cellular characteristics and responses [80]. In our study, a broader range of cell lines was evaluated to address this variability. Each cell line has unique genetic and phenotypic properties that influence how it interacts with bioactive compounds, potentially leading to variable results in assays [81]. Including multiple cell types in the study provided a more comprehensive understanding of the compound’s effects across various cellular environments, ensuring that the findings are more robust and relevant in potential therapeutic applications [82].

## 4. Conclusions

Studying the hemolytic, anti-hemolytic, antioxidant, and cytotoxic effects of natural products such as traditionally used plants is a complex field that requires careful consideration of factors such as dosage, mode of administration, and the specific context of use.

In the present research, we report an overview of the positive in vitro effects of some of the plants commonly used in Mexico in a variety of cell lines. We also report the possible side effects that they can cause in healthy cell cultures (in vitro) caused by an increase in their dose and concentration.

Continued research in this area promises to broaden our understanding of the safety of these bioactive natural products and explore their therapeutic potential in various bio-medical applications.

## Figures and Tables

**Table 1:** Taxonomic identification of the plants and yield extraction percentage.

Voucher	Family	Taxonomical Identification	Traditional Name	Part	Yields (%)
11002	Crassulaceae	*Kalanchoe daigremontiana* Raym.-Hamet & H.Perrier	Aranto	L	9.96
29127	Papaveraceae	*Argemone mexicana* L.	Chicalote	L	11.3
30642	Anacardiaceae	*Amphipterygium adstringens* (Schltdl.) Standl.	Cuachalalate	B	38.4
30643	Compositae	*Artemisia ludoviciana* Nutt.	Estafiate	L	19.0
30644	Poaceae	*Cymbopogon citratus* (DC.) Stapf.	Zacate limón	L	23.0
30646	Compositae	*Heterotheca inuloides* Cass.	Arnica	F	21.0
30647	Celastraceae	*Semialarium mexicanum* (Miers) Mennega	Cancerina	B	11.0
30648	Euphorbiaceae	*Jatropha dioica* Sessé	Dragon’s Blood	R	16.0
30649	Acanthaceae	*Justicia spicigera* Schltdl.	Muicle	L	13.2
30650	Zygophyllaceae	*Larrea tridentata* (Sessé & Moc. ex DC.) Coville	Gobernadora	L	13.2
30651	Leguminosae	*Mimosa tenuiflora* (Willd.) Poir.	Tepezcohuite	B	10.8
30652	Compositae	*Psacalium peltatum* (Kunth) Cass.	Matarique	L	10.9
30653	Compositae	*Pseudognaphalium obtusifolium* (L.) Hilliard & B.L.Burtt.	Gordolobo	L	17.0
30654	Rutaceae	*Ruta chalepensis* L.	Rude	R	19.4
30655	Smilacaceae	*Smilax aspera* L.	Zarzaparrilla	L	13.1
30656	Compositae	*Tagetes lucida* Cav.	Yerbaniz	B	20.6

L: Leaves; B: barks; F: flowers; R: roots; %: percentage of extraction yield.

**Table 2:** Phytochemical screening of extracts.

	Chemical Group									
Plant Extract	Alk	Carb	Cm	Db	Flv	Qn	Sp	Sl	St	Tn
*K. daigremontiana*	+	+	−	+	+	−	+	−	+	−
*A. mexicana*	+	−	+	+	+	−	−	+	+	−
*A. adstringens*	−	+	+	+	+	+	−	+	+	+
*A. ludoviciana*	−	+	+	+	+	+	−	+	+	+
*C. citratus*	−	+	+	+	+	−	−	−	+	−
*H. inuloides*	−	+	+	+	+	−	−	−	+	+
*S. mexicanum*	−	+	+	+	−	+	−	+	−	−
*J. dioica*	−	+	+	+	−	+	−	−	+	−
*J. spicigera*	−	+	+	+	−	+	−	−	−	+
*L. tridentata*	−	−	+	+	+	+	−	+	+	+
*M. tenuiflora*	−	−	+	+	+	+	−	+	+	+
*P. peltatum*	−	+	+	+	+	−	−	−	+	+
*P. obtusifolium*	−	+	+	+	+	+	+	+	+	+
*R. chalepensis*	+	+	+	+	+	−	−	+	+	+
*S. aspera*	−	+	+	+	−	+	−	+	−	−
*T. lucida*	−	+	+	+	+	−	−	+	−	+

Alk: alkaloids, Carb: carbohydrates, Cm: coumarins, Db: double bonds, Flv: flavonoids, Qn: quinones, Sp: saponins, Sl: sesquiterpene—lactones, St: sterols, Tn: tannins, +: positive reaction, −: negative reaction.

**Table 3:** Hemolytic and anti-hemolytic assays of Mexican plant extracts.

Plant Extract	Hemolysis	Anti-Hemolytic	SI
	IC_50_ in µg/mL		
*K. daigremontiana*	671.81 ^d^	12.33 ^ab^	54.48
*A. mexicana*	973.88 ^g^	21.06 ^b^	46.24
*A. adstringens*	182.87 ^a^	5.35 ^a^	34.18
*A. ludoviciana*	825.67 ^f^	15.93 ^ab^	51.83
*C. citratus*	558.62 ^c^	13.17 ^ab^	42.42
*H. inuloides*	723.80 ^e^	8.09 ^a^	89.47
*S. mexicanum*	>1500 ^†^	15.29 ^ab^	>98.10
*J. dioica*	613.54 ^cd^	67.67	9.07
*J. spicigera*	>1500 ^†^	32.05 ^c^	>46.80
*L. tridentata*	550.77 ^c^	150.45 ^d^	3.66
*M. tenuiflora*	>1500 ^†^	15.34 ^ab^	>97.78
*P. peltatum*	>1500 ^†^	10.54 ^a^	>142.31
*P. obtusifolium*	>1500 ^†^	20.68 ^b^	>72.53
*R. chalepensis*	870.75 ^f^	15.45 ^ab^	56.36
*S. aspera*	387.94 ^b^	11.25 ^ab^	34.48
*T. lucida*	891.79 ^f^	9.03 ^a^	98.76

Different letters within the same column are significantly different by Tukey’s test. ^†^ As IC_50_ was above 1500 µg/mL, these values were not considered for the Tukey analysis.

**Table 4:** Antioxidant activities of Mexican plant extracts.

Plant Extract	DPPH Assay
	IC_50_ in µg/mL
*K. daigremontiana*	699.05 ^d^
*A. mexicana*	655.39 ^c^
*A. adstringens*	700.50 ^d^
*A. ludoviciana*	949.73 ^f^
*C. citratus*	1011.64 ^fg^
*H. inuloides*	897.79 ^e^
*S. mexicanum*	>1500 ^†^
*J. dioica*	>1500 ^†^
*J. spicigera*	>1500 ^†^
*L. tridentata*	665.41 ^c^
*M. tenuiflora*	547.66 ^b^
*P. peltatum*	520.52 ^b^
*P. obtusifolium*	528.67 ^b^
*R. chalepensis*	859.85 ^e^
*S. aspera*	936.50 ^f^
*T. lucida*	578.62 ^b^
Vit. C	9.57 ^a^

Different letters within the same column are significantly different by Tukey’s test. ^†^ As IC_50_ was above 1500 µg/mL, these values were not considered for the Tukey analysis.

**Table 5:** Cytotoxic activities of Mexican plant extracts.

Plant Extract	IC_50_ in µg/mL	
	Vero Cells	PBMC Cells
*K. daigremontiana*	107.13 ^b^	ND
*A. mexicana*	200.17 ^c^	398.45 ^c^
*A. adstringens*	146.55 ^bc^	ND
*A. ludoviciana*	188.75 ^bc^	744.56 ^e^
*C. citratus*	664.03 ^f^	287.07 ^a^
*H. inuloides*	ND	1076.18 ^g^
*S. mexicanum*	569.34 ^e^	ND
*J. dioica*	ND	1317.13 ^h^
*J. spicigera*	ND	ND
*L. tridentata*	197.93 ^b^	ND
*M. tenuiflora*	467.59 ^d^	ND
*P. peltatum*	54.91 ^a^	670.30 ^d^
*P. obtusifolium*	61.98 ^a^	745.40 ^e^
*R. chalepensis*	802.83 ^h^	346.84 ^b^
*S. aspera*	589.46 ^e^	391.60 ^c^
*T. lucida*	780.62 ^g^	848.82 ^f^

Different letters within the same column are significantly different by Tukey’s test. ND: Not determined because it promotes cell proliferation.

## Data Availability

The datasets generated or analyzed during the present study are available upon request from the corresponding author.
